# Gas Sensors and Semiconductor Nanotechnology

**DOI:** 10.3390/nano12081322

**Published:** 2022-04-12

**Authors:** János Mizsei

**Affiliations:** Department of Electron Devices, Budapest University of Technology and Economics, Műegyetem rkp. 3, H-1111 Budapest, Hungary; mizsei.janos@vik.bme.hu

Solid-state semiconductor gas sensors have been attracting a great deal of attention for over two decades, due to their importance in gas analysis and safety applications. The chemical sensitivity of a semiconductor surface serves as a way to transduce the chemical information about the surfaces into an electrical signal for gas-sensing applications.

Sensor technology development has a long history [1]. It includes thick-film and thin-film technology, and, recently, semiconductor nanotechnology. Size-dependent physical properties are very important in the theory and construction of sensor devices, and metal and semiconductor nanoparticles are basic components of older and even recently used gas-sensitive materials.

This Special Issue of *Nanomaterials* will attempt to show some items from the gas sensor technology, and nano-sized structures for gas sensor applications.

The first article (Orientation Ordering and Chiral Superstructures in Fullerene Monolayer on Cd (0001)) [2] is not strongly related to the gas sensor subject. However, the fullerene thin film interaction with the Cd surface has been investigated by STM, including spectroscopy for the HOMO-LUMO energy gap. The virtuous use of experimental techniques resulted in high-quality images of Cd-Fullerene structures. The results are of great importance to the carbon-based nanotechnology, nano-devices, and nanomaterials.

All other articles [3,4,5,6,7,8] are related to different nanostructured semiconductor gas sensor materials for numerous gases (see lists below) with different types of sensor constructions, such as the surface acoustic wave substrate, Taguchi type (ceramic tube substrate with heater inside), or just simple heated insulator plate substrate (oxidized silicon, or others, for example Al_2_O_3_).
**Material****Technology****Gas Sensitivity**mesoporous AlO(OH)sol-gel and spin-coatingNH_3_SnO_2_–NiO nanoneedleshydrothermal synthesisNO_2_α-Fe_2_O_3_ nanoparticlesin situ corrosion method of scoroditexyleneNiO-ZnO nanorodshydrothermal/sol-gelH_2_SWO_3_–SnO_2_ composite nanorodhydrothermal/sputteringacetoneSnO_2_, Pd/SnO_2_, Au/SnO_2_, AuPd/SnO_2_ nanocompositesflame spray pyrolysisC_3_H_8_, CO, CH_4_, H_2_, NO_2_, NH_3_, acetone.

Articles [3,4,5,6,7,8] detail the experimental technique, analytical methods, measurement methods, and results in sufficient depth. Developments are successful concerning sensor properties: all gas sensors produced significant selectivity and sensitivity to the given gases.

Generally, it can be concluded that nanotechnology adds some extra possibilities to conventional technologies and materials; see more in [9].
